# The stroke seesaw: unveiling the enigma of stuttering acute ischemic stroke

**DOI:** 10.1097/MS9.0000000000002780

**Published:** 2025-01-31

**Authors:** Abuoma C. Ekpendu, Puneet Prakash, Chad K. Brands

**Affiliations:** Internal Medicine, AdventHealth Sebring, Sebring, Florida, USA

**Keywords:** acute ischemic stroke, case report, stuttering, tissue plasminogen activator, transient ischemic attack

## Abstract

**Introduction:**

Beyond the familiar face of the acute ischemic stroke, lies the unexpected display of symptoms challenging our preconceptions and beckoning us to explore the intriguing nuances that lie beneath the surface.

**Presentation of case:**

We introduce an unusual case of an older adult who presented to our institution with repetitive focal neurologic deficits (FND). Initial investigation was negative for any brain hemorrhage or infarcts. Repeat investigation during readmission showed findings of brain infarct for which appropriate treatment was provided. The fluctuating pattern of FND is what we refer to as a stuttering presentation of stroke.

**Discussion:**

Our case brings attention to stuttering, an atypical presentation of ischemic stroke, not previously well described in medical literature. Our case demonstrates that stuttering stroke presentations may imitate more short-lived neurologic events such as transient ischemic attacks.

**Conclusion:**

All the patient events throughout the entirety of their time course should be thoroughly reviewed so that thrombolytics can then be considered in the earliest window of opportunity for patients with stuttering stroke presentations to prevent disability due to ischemic stroke.

## Introduction

Stroke stands as a leading cause of mortality globally, casting a significant public health burden worldwide^[1]^. The spectrum of stroke includes transient ischemic attacks (TIAs), distinguished by symptoms persisting for less than 24 hours with absence of permanent cerebral infarction. However, the diagnostic landscape can be marked by atypical presentations, challenging clinicians evaluating patients presenting with a spectrum of neurologic signs and symptoms. Ischemic stroke takes the forefront of this spectrum, along the intricate network of vascular disruptions resulting in neurological consequences seen clinically.

Our patient’s presentation, characterized by intermittent symptoms over a protracted time course, underscores the need for further exploration of the underlying pathology and nuanced understanding and in both diagnosis and treatment.

This case report has been reported in line with the SCARE 2023 Criteria^[2]^.

## Presentation of case

A 70-year-old male with a past medical history of hypertension, hyperlipidemia, gout, psoriasis, and no prior stroke history, presented to our emergency department (ED) with difficulty speaking, bilateral lower extremity weakness, and left facial droop that began 1.5 hours prior to ED arrival. He had smoked 1.5 packs of cigarettes per day since he was a teenager. He consumed about 6 beers per day and denied any recreational drug use. Vital signs in the ED revealed a heart rate of 79, respiratory rate of 18, blood pressure of 186/106, temperature of 90 F, and oxygen saturation of 97% on room air. Upon initial presentation and physical, his symptoms had resolved, and the initial National Institutes of Health Stroke Scale score (NIHSS) was 0. Therefore, tissue plasminogen activator (tPA) therapy was not provided.

**Figure 1.**
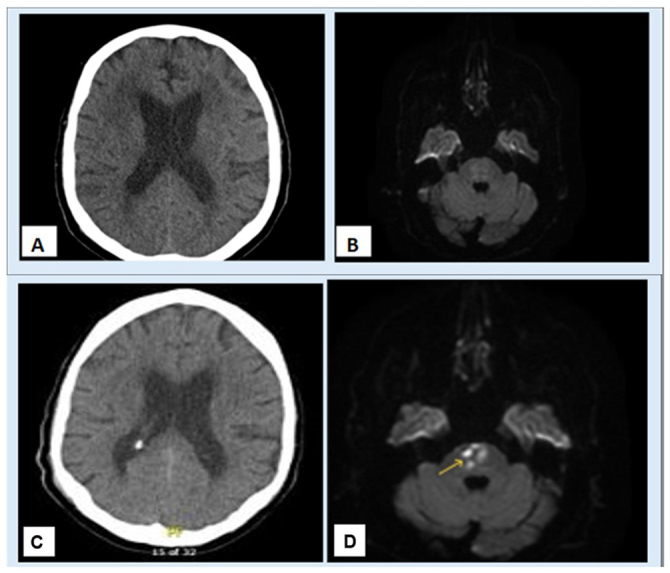
(A) Non-contrast computed tomography of the brain at initial presentation, showing no hemorrhage. (B) Magnetic resonance imaging (MRI) of the brain at initial presentation, showing no acute infarct. (C) Non-contrast computed tomography of the brain obtained during second presentation, showing no acute hemorrhage. (D) DWI MRI of the brain obtained during second presentation, showing acute pontine infarct.

Laboratory studies were significant for total cholesterol level of 289 mg/dL, triglycerides level of 495 mg/dL, white blood cell count (WBC) of 6.26 × 10^3^ per microliter (µL), BUN/Cr ratio of 15.6, and blood glucose level of 123. Chest X-ray showed no acute lung abnormality. Computed tomography (CT) scan of the brain without contrast showed no acute intracranial abnormality (Fig. 1). Magnetic resonance imaging (MRI) of the brain showed no acute infarcts (Figure 1). Bilateral carotid Doppler ultrasound showed suspected 50–69% stenosis in the right internal carotid artery (ICA) and less than 50% stenosis suspected in the left ICA. Further diagnostic imaging with a CT angiogram of the head and neck showed atherosclerotic disease involving the bilateral carotid bulbs, proximal ICA, and intracranial internal carotids but with no hemodynamically significant stenosis. Electrocardiogram (ECG) upon presentation showed sinus rhythm. Echocardiogram with bubble study revealed estimated ejection fraction of 55–60%, a sclerosis of the aortic valve with peak velocity of 1.8 m/s, and no septal defect or patent foramen ovale identified.

**Figure 2.**
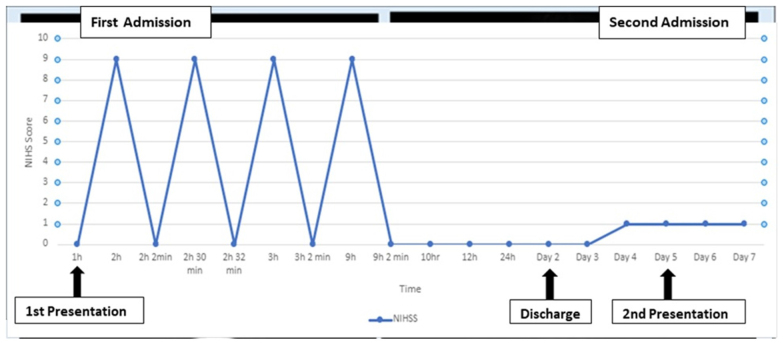
Stuttering pattering of stroke symptoms/National Institutes of Health Stroke Scale score (NIHSS). The first half of this graph depicts the stuttering pattern of focal neurologic deficits during the first hospital admission. Symptoms occurred about every 20–30 minutes for a duration of at least 30–45 seconds (some episodes lasted up to 2 minutes or more), with a return to baseline neurologic status. NIHSS was up to 9 during each episode then down to 0 when resolved. The second half of Fig. 1 shows a stable pattern of symptoms during the second hospital admission.

Subsequently in the ED, the patient had multiple recurrent episodes of left-sided extremity weakness, left facial droop, and dysarthria occurring about every 20–30 minutes. Each episode lasted at least about 30–45 seconds followed by a return to baseline neurologic status. His NIHSS went up to 9 during each episode then down to 0 when these symptoms resolved (Fig. 2). Of note, sensation was grossly intact, there was no hearing loss, or ophthalmoplegia. During the next several hours of the hospital course, the patient again had another episode of similar focal neurologic deficits (FND).

Our patient’s initial negative CT and MRI, coupled with resolution of symptoms in less than 24 hours, led to the initial diagnosis of TIA. Vascular surgery recommended atherosclerotic cardiovascular disease (ASCVD) risk factors modification; therefore, endarterectomy was not offered. Patient was treated appropriately using dual antiplatelet therapy (DAPT) with aspirin and clopidogrel, and also a high dose statin. He recovered well and was discharged without symptoms of neurologic deficits on hospital day 3.

**Figure 3.**
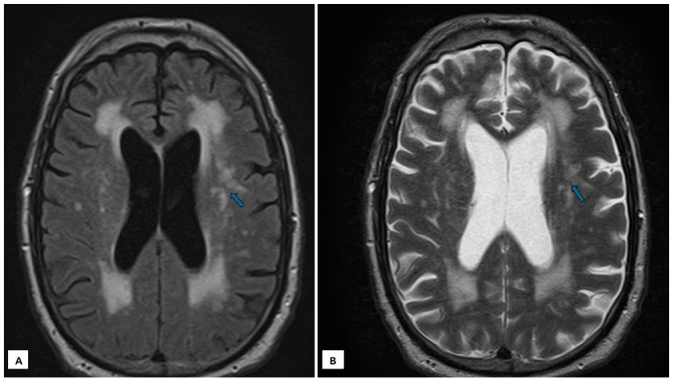
(A) Flair magnetic resonance imaging (MRI) of the brain showing cerebral small vessel disease. (B) T2 MRI of the brain showing cerebral small vessel disease.

The patient returned to the ED 3 days after hospital discharge with progressive slurred speech and bilateral lower extremity weakness. This time, the symptoms were sustained. Repeat CT of the head showed no intracranial hemorrhage (Figure 1). Again, the patient was not considered for tPA due to ongoing symptoms lasting multiple days. Furthermore, repeat MRI of the brain at this time with and without contrast revealed an acute infarction located in the mid-pons (Figure 1) along with extensive small vessel disease (Figures 3). Further diagnostics with Magnetic Resonance Angiogram (MRA) of the head and neck revealed limited flow within the right external carotid artery concerning for subtotal occlusion without appreciable vertebrobasilar stenosis or flow-limiting stenosis of the internal carotid or cerebral arteries. There were no cerebral microhemorrhages identified on imaging.

The patient was continued on aspirin, clopidogrel, and high dose statin therapy. On hospital day 8, he was discharged with residual dysarthria to inpatient rehabilitation with scheduled outpatient follow up. Hospital course is depicted in Table 1.

**Table 1:** Hospital course

Days	Day 0	Day 1	Day 2	Day 0 (readmission—3 days after discharge)	Days 1–7
Timeline + symptoms	0 hour (h) (at home): bilateral leg weakness, dysarthria	24–36 h (inpatient): symptoms resolved within 24 h	48 h: Back to Baseline neurologic function	0 h of symptom recurrence (at home): dysarthria	Dysarthria remained
Symptom progression	1–1.5 h (ED): NIHSS 0	Treated with dual antiplatelet therapy (aspirin + Plavix) and high dose statin	Discharged without focal neurologic deficits	0–72 hours (at home): progressive dysarthria with bilateral lower extremity weakness	Improved strength and activity tolerance
Symptom progression	2–10 h (Inpatient): episodes of left sided weakness, facial droop, dysarthria, about every 20 minutes for 30–45 seconds			About 84 h (ED): Persisting dysarthria bilateral lower extremity weakness, left facial drop	Discharged to inpatient rehabilitation on hospital day 8 on Dual antiplatelet therapy and high dose statin
	11–12 h: Last episode with similar neurologic symptoms				

Showing timeline of events from presentation through discharge.

## Discussion

The 2022 update on heart disease and stroke statistics revealed that in the United States, approximately 795 000 people experience a stroke (new or recurrent) with approximately 610 000 and 185 000 being first and recurrent attacks, respectively. An estimated 7.6 million Americans aged 20 years and above have had a stroke. Eighty-seven percent (87%) of all strokes are ischemic, while 10% and 3% of all strokes are attributed to intracerebral and subarachnoid hemorrhages, respectively^[3]^. In the United States, stroke is the fifth leading causes of death and the second leading cause of death globally^[1,4]^.

Both TIA and stroke are clinical syndromes occurring due to vascular brain injury of varying mechanisms with the former lasting less than 24 hours without permanent cerebral infarction, and the latter lasting 24 hours or more. However, applying these definitions to clinical practice may lead to diagnostic delay as clinical presentations of both entities may be atypical and recurrent. Therefore, experts recommend that time-based definition for strokes should be considered less rigid^[5]^.

Small penetrating vessels are most affected by chronic hypertension (leading modifiable risk factor for stroke), resulting in cerebral small vessel disease, which would cause hyperplasia of the tunica media and fibrinoid material deposition causing narrowing and occlusion of the small vessel lumen thereby predisposing to ischemia^[6,7]^. Cardioembolic strokes typically occur suddenly, progress quickly and recover rapidly. Thrombotic and lacunar stroke symptoms regularly have fluctuating FNDs, which may progress in a stuttering pattern and may have periods of improvement^[7,8]^. Lacunar strokes occur due to obstruction of small penetrating arteries and are associated with worsening motor deficits after hospital admission^[5,8]^.

The differential diagnoses of stuttering or fluctuating FND in this case include demyelinating disease, unruptured brain arteriovenous malformation (BAVM), focal seizures, masses, cerebral amyloid angiopathy (CAA), functional neurologic disorder^[9-12]^. In our case, the physical exam findings, the lack of supporting history, absence of any other brain lesions or impaired consciousness and the presence of infarction, makes focal seizure, BAVM, demyelinating disease, functional neurologic disorder, CAA or masses less likely^[9-12]^.

Although literature regarding the mechanism of stuttering stroke is scarce, some studies have suggested cerebral small vessel disease and step-by-step blockage of the branches of small perforating vessels by large intracranial arterial lesions, as possible mechanisms^[8,13]^. Furthermore, the stroke warning syndrome (SWS) has been described as a form of recurring TIA carrying a high risk of infarction. It involves recurring sensorimotor symptoms affecting the leg, arm and face and occurs within 7 days after a TIA. Postulated mechanisms include small vessel disease, periinfarct depolarization, hemodynamic instability, and artery to artery embolism. A SWS involving the middle and anterior cerebral artery is called capsular warning syndrome (CWS) while involvement of posterior circulation is called pontine warning syndrome ^[14]^.

The fluctuating pattern of FND interspersed with periods of return to baseline neurologic function, is what we refer to as stuttering. Our patient’s stuttering pattern of stroke symptoms and time course point toward a lacunar stroke possibly from occlusion of small penetrating arteries in the posterior circulation.

There are no specific guidelines regarding diagnosis and treatment of stuttering stroke. For strokes in general, non-contrast CT scan of the brain is usually the initial imaging obtained to rule out intracerebral hemorrhage. Infarctions are frequently missed on CT scan therefore MRI of the brain is more sensitive and can detect an acute infarction within minutes of stroke onset. ECG and troponin should be obtained since stroke is often associated with coronary artery disease. Secondary stroke prevention is recommended for strokes. This involves treatment with high dose statin, DAPT for 21 days followed by aspirin monotherapy. Mechanical thrombectomy should be considered in patients with large vessel occlusions in the first 6 hours of stroke onset. Intravenous alteplase (tPA) should be considered within 3–4.5 hours of symptom onset for appropriate patients with FND.^[15,16]^

## Conclusion

Our case brings attention to an atypical presentation of stroke, not previously well described in the medical literature. Stuttering stroke presentations may imitate more short-lived neurologic events such as TIAs. Therefore, we suggest that all events along the entirety of the patient’s course be reviewed quickly and thoroughly at presentation and throughout the hospital course. Thrombolytic therapy can then be considered in the earliest window of therapeutic opportunity to prevent disability. Further research is needed to determine the mechanism and appropriate management of stuttering strokes.

## Data Availability

Not applicable.
